# Supplementary material for: Drug repurposing for aging research using model organisms
Source: Aging Cell. 2017 Jun 16;16(5):1006–15. doi: 10.1111/acel.12626 (PMC5595691; doi:10.1111/acel.12626)
Supplement: Supplementary file 7 — Data S1 Zip‐Archive of all report cards. [file ACEL-16-1006-s007.zip › RC_KSA.pdf]

KSA

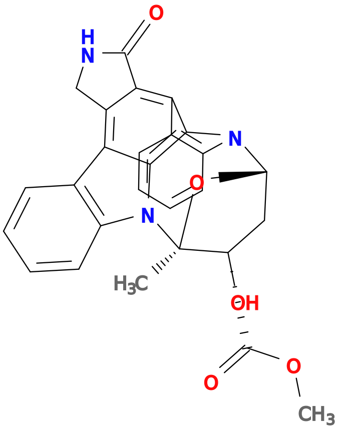

Database identifiers

ChEMBLCompound	CHEMBL281948
DrugBank	DB02152
CHEBI	43616
ZINC	ZINC03872984
eMolecules	29547198
eMolecules	30488043
eMolecules	476106

Ranking

	Rank	Score
Drosophila	132/697	0.762
C. elegans	125/591	0.302

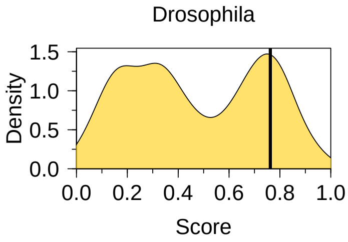

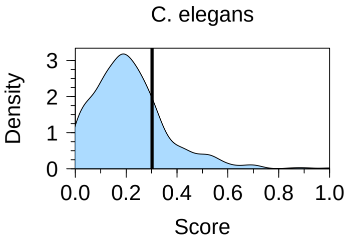

	Ageing implication	Domain conservation	Binding site conservation	Binding affinity	Bioavailability	Lipinski	Promiscuity	Purchasability	Drug approval	**Total**
Drosophila	0.81	0.965	0.876	0.959	(0.9)	0.0	-0.004	0.1	0.075	0.762
C. elegans	0.81	0.94	0.758	0.959	0.235	0.0	-0.004	0.1	0.075	0.302

Names

- **K-252a**
- Antibiotic K 252a
- Antibiotic SF 2370
- K252a
- Sf 2370

Roles

EC 2.7.11.13 (protein kinase C) inhibitor, antimicrobial agent, tropomyosin-related kinase B receptor antagonist, bacterial metabolite

Status

Approved drug (according to ChEMBL)	No
Classification (according to DrugBank)	experimental
Number of Rule of 5 violations	0
Binding affinity to original target in log units (RF-Score prediction)	8.16
Burns *C. elegans* bioavailability prediction	-2.92

Compound Target Characteristics

Inhibitor of nuclear factor kappa-B kinase subunit beta

Best gene implication in ageing for this target family came from gene Q3U141 via mapping the annotation from Ensembl ENSMUSG00000031537 via mapping the annotation from EntrezGene 16150 via mapping the annotation from GenAgeModels 1811 annotated in GenAge release 17.

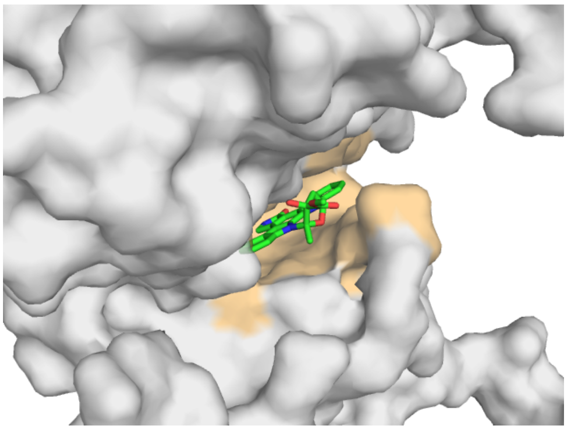

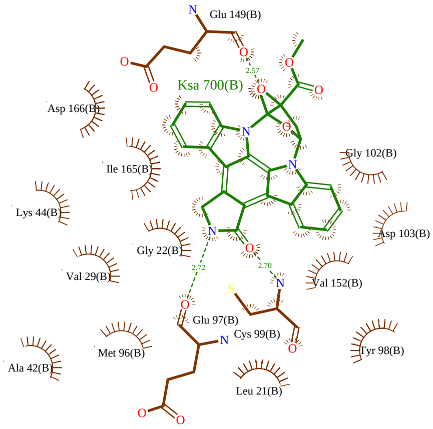

protein	amino acids contacts (binding site)
PDB:4kik:chainA:O14920	L G T V A K M E Y C G D E N V I D
PDB:4kik:chainB:O14920	L G T V A K M E Y C G D E N V I D
tr:E5RGW5:E5RGW5_HUMAN	L G T V A K M E Y C G D E N V I D
tr:G3V105:G3V105_HUMAN	L G T V A K M E Y C G D E N V I D
sp:O14920:IKKB_HUMAN	L G T V A K M E Y C G D E N V I D
tr:G3V8H5:G3V8H5_RAT	L G T V A K M E Y C G D E N V I D
tr:Q3U141:Q3U141_MOUSE	L G T V A K M E Y C G D E N V I D
sp:O88351:IKKB_MOUSE	L G T V A K M E Y C G D E N V I D
tr:Q5D0E0:Q5D0E0_MOUSE	L G T V A K M E Y C G D E N V I D
sp:Q9VEZ5:IKKB_DROME	L G E V A K L E Y C G D D N V T D

	whole protein		domain-based		contact-based	
protein	ident	simil	ident	simil	ident	simil
PDB:4kik:chainA:O14920	1.0	1.0	1.0	1.0	1.0	1.0
PDB:4kik:chainB:O14920	1.0	1.0	1.0	1.0	1.0	1.0
tr:E5RGW5:E5RGW5_HUMAN	0.5	0.5	1.0	1.0	1.0	1.0
tr:G3V105:G3V105_HUMAN	0.68	0.72	1.0	1.0	1.0	1.0
sp:O14920:IKKB_HUMAN	1.0	1.0	1.0	1.0	1.0	1.0
tr:G3V8H5:G3V8H5_RAT	0.91	0.97	0.96	0.99	1.0	1.0
tr:Q3U141:Q3U141_MOUSE	0.52	0.57	0.97	0.99	1.0	1.0
sp:O88351:IKKB_MOUSE	0.92	0.98	0.97	0.99	1.0	1.0
tr:Q5D0E0:Q5D0E0_MOUSE	0.92	0.98	0.97	0.99	1.0	1.0
sp:Q9VEZ5:IKKB_DROME	0.19	0.59	0.36	0.74	0.76	0.88

ird5 (FBgn0024222) associated phenotypes

immune response defective, partially lethal - majority die

(Information from FlyBase)

ird5 (UniProt:Q9VEZ5) annotation

Function: Required for the activation of the NF-kappa-B factor Relish (Rel) by acting as an essential signaling component in transmitting the lipopolysaccharide (LPS) signal leading to cact degradation, which is required for direct activation of Rel. Phosphorylates inhibitors of NF-kappa-B (cact) thus leading to the dissociation of the inhibitor/NF-kappa-B complex and ultimately the degradation of the NF-kappa-B inhibitor. Essential for antibacterial immune response. (PubMed:10636911, PubMed:11018014, PubMed:11156609).

Subunit: Interacts with key to form the I-kappa-B kinase complex. In vitro, interacts with cact. (PubMed:10636911, PubMed:11018014).

Subcellular location: Cytoplasm ECO:0000250.

Developmental stage: Expressed both maternally and zygotically throughout development. Highest expression is in early embryos and third larval instar. (PubMed:10636911).

Ptm: Autophosphorylated; upon LPS stimulation it is transiently activated, and can be autophosphorylated. (PubMed:18327897).

(Information from UniProt)

Hepatocyte growth factor receptor

Best gene implication in ageing for this target family came from gene Q2IBC7 via mapping the annotation from RGD 3082 annotated in RGD 2014-03-11. Annotation GO subterm of 7568 (aging) was Inferred from Expression Pattern

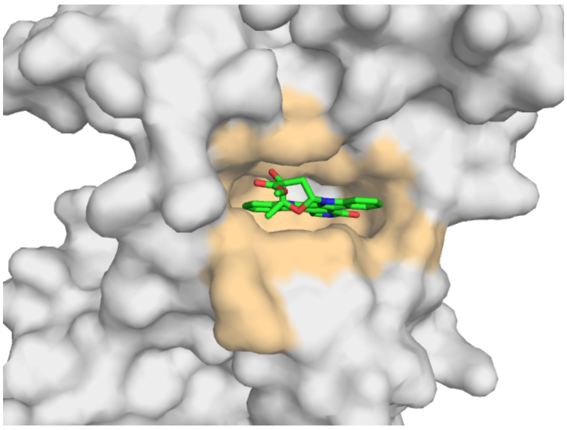

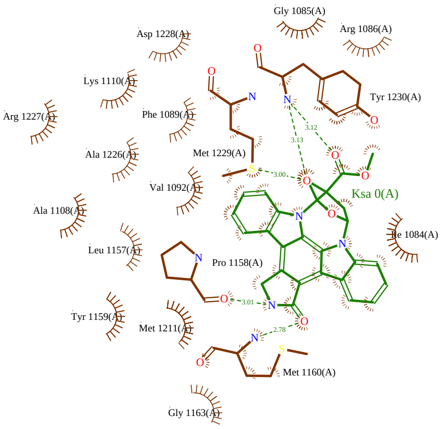

protein	amino acids contacts (binding site)
PDB:1r0p:chainA:P08581	I G R F V A K L P Y M G M A R D M Y
tr:B4DLF5:B4DLF5_HUMAN	I G R F V A K L P Y M G M A R D M Y
sp:P08581:MET_HUMAN	I G R F V A K L P Y M G M A R D M Y
tr:Q2IBC7:Q2IBC7_RAT	I G R F V A K L P Y M G M A R D M Y
tr:F8VQL0:F8VQL0_MOUSE	I G R F V A K L P Y M G M A R D M Y
tr:Q6AHP3:Q6AHP3_CAEEL	I G Q Y V V K T E Y M G L C R K V -
tr:H1AGA1:H1AGA1_CAEEL	I G Q Y V V K T E Y M G L C R K V -
tr:H2KZU7:H2KZU7_CAEEL	I G Q Y V V K T E Y M G L C R K V -

	whole protein		domain-based		contact-based	
protein	ident	simil	ident	simil	ident	simil
PDB:1r0p:chainA:P08581	0.99	1.0	0.99	1.0	1.0	1.0
tr:B4DLF5:B4DLF5_HUMAN	0.69	0.69	1.0	1.0	1.0	1.0
sp:P08581:MET_HUMAN	1.0	1.0	1.0	1.0	1.0	1.0
tr:Q2IBC7:Q2IBC7_RAT	0.88	0.96	0.98	1.0	1.0	1.0
tr:F8VQL0:F8VQL0_MOUSE	0.89	0.96	0.99	1.0	1.0	1.0
tr:Q6AHP3:Q6AHP3_CAEEL	0.13	0.4	0.41	0.78	0.44	0.56
tr:H1AGA1:H1AGA1_CAEEL	0.13	0.4	0.41	0.78	0.44	0.56
tr:H2KZU7:H2KZU7_CAEEL	0.13	0.4	0.41	0.78	0.44	0.56

Dual specificity mitogen-activated protein kinase kinase 1

Best gene implication in ageing for this target family came from gene Q02750 annotated in UniProt release 2014_02. Annotation GO subterm of 7568 (aging) was Inferred from Mutant Phenotype

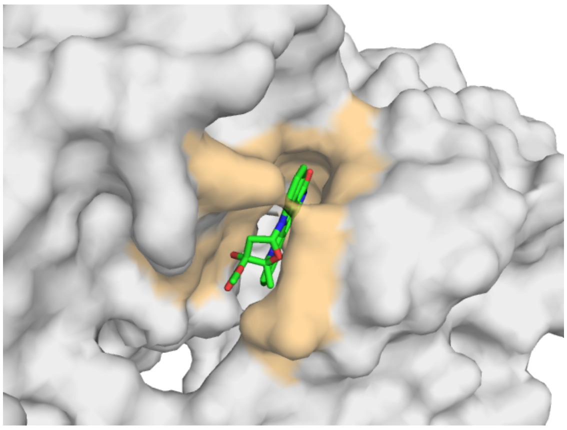

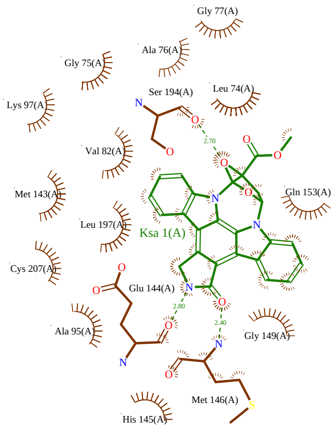

protein	amino acids contacts (binding site)																
PDB:3eqf:chainA:Q02750	L	G	A	G	V	A	K	M	E	H	M	G	Q	S	N	L	C
sp:Q02750:MP2K1_HUMAN	L	G	A	G	V	A	K	M	E	H	M	G	Q	S	N	L	C
tr:A4QPA9:A4QPA9_HUMAN	L	G	A	G	V	A	K	M	E	H	M	G	Q	S	N	L	C
sp:Q01986:MP2K1_RAT	L	G	A	G	V	A	K	M	E	H	M	G	Q	S	N	L	C
tr:Q3TMJ8:Q3TMJ8_MOUSE	L	G	A	G	V	A	K	M	E	H	M	G	Q	S	N	L	C
sp:P31938:MP2K1_MOUSE	L	G	A	G	V	A	K	M	E	H	M	G	Q	S	N	L	C
sp:Q24324:DSOR1_DROME	L	G	S	G	V	A	K	M	E	Y	M	G	L	S	N	L	C
sp:Q10664:MEK2_CAEEL	L	G	H	G	V	A	K	M	E	Y	M	L	I	S	N	L	C
sp:P08018:PBS2_YEAST	L	G	H	G	V	A	K	M	E	Y	M	G	K	T	N	L	C

	whole protein		domain-based		contact-based	
protein	ident	simil	ident	simil	ident	simil
PDB:3eqf:chainA:Q02750	1.0	1.0	1.0	1.0	1.0	1.0
sp:Q02750:MP2K1_HUMAN	1.0	1.0	1.0	1.0	1.0	1.0
tr:A4QPA9:A4QPA9_HUMAN	1.0	1.0	1.0	1.0	1.0	1.0
sp:Q01986:MP2K1_RAT	0.99	1.0	1.0	1.0	1.0	1.0
tr:Q3TMJ8:Q3TMJ8_MOUSE	0.99	1.0	1.0	1.0	1.0	1.0
sp:P31938:MP2K1_MOUSE	0.99	1.0	1.0	1.0	1.0	1.0
sp:Q24324:DSOR1_DROME	0.6	0.82	0.75	0.93	0.82	0.83
sp:Q10664:MEK2_CAEEL	0.48	0.74	0.62	0.88	0.76	0.76
sp:P08018:PBS2_YEAST	0.2	0.36	0.45	0.74	0.76	0.84

Dsor1 (FBgn0010269) associated phenotypes

dominant, lethal - all die before end of pupal stage, neuroanatomy defective, polyphasic, some die during immature adult stage, some die during pupal stage

(Information from FlyBase)

Dsor1 (UniProt:Q24324) annotation

Function: Required downstream of Raf in the sevenless (sev), torso (tor), and Drosophila EGF receptor homolog (DER) signal transduction pathways. Involved in both positive regulation (at the posterior terminus) and negative regulation (at the anterior domain) of tll, as in other terminal class gene products, maybe via the ERK-A kinase. (PubMed:8381718).
Developmental stage: Expressed both maternally and zygotically. (PubMed:8381718).
Ptm: Phosphorylation on Ser/Thr by MAP kinase kinase kinases regulates positively the kinase activity.

(Information from UniProt)

mek-2 (WBGene00003186) associated phenotypes
AWA odorant chemotaxis defective, AWC odorant chemotaxis defective, bacterially unswollen, constipated, germ cell arrest, germ nuclei rachis, lethal, metabolic pathway variant, no oocytes, pachytene region organization variant, pathogen susceptibility increased, rod like larval lethal, sterile, vulval cell lineage variant, vulvaless

(Information from WormBase)

mek-2 (UniProt:Q10664) annotation
Function: Functions in the let-60 Ras signaling pathway; acts downstream of lin-45 raf kinase, but before the sur-1/mpK-1 gene product in controlling vulval cell differentiation.
Enzyme regulation: Activated by tyrosine and threoninephosphorylation catalyzed by MAP kinase kinase kinases.

(Information from UniProt)
